# The Light-Dosimeter: A new device to help advance research on the non-visual responses to light

**DOI:** 10.1177/14771535221147140

**Published:** 2023-02-13

**Authors:** JR Stampfli, B Schrader, C di Battista, R Häfliger, O Schälli, G Wichmann, C Zumbühl, P Blattner, C Cajochen, R Lazar, M Spitschan

**Affiliations:** a Lucerne School of Engineering and Architecture, Horw, Switzerland; b Federal Institute of Metrology (METAS), Bern-Wabern, Switzerland; c Centre for Chronobiology, Psychiatric Hospital of the University of Basel (UPK), Basel, Switzerland; d Research Platform Molecular and Cognitive Neurosciences, University of Basel, Basel, Switzerland; e Department of Experimental Psychology, University of Oxford, Oxford, UK

## Abstract

This article describes the development of a device to investigate the non-visual responses to light: The Light-Dosimeter (lido). Its multidisciplinary team followed a user-centred approach throughout the project, that is, their design decisions focused on researchers’ and participants’ needs. Together with custom-made mountings and the software Lido Studio, the lidos provide researchers with a holistic solution to record participants’ light exposure in the near-corneal plane in laboratory settings and under real-world conditions. Validation measurements with commercial equipment were deemed satisfying, as was the combining with data from other devices. The handling of the lidos and mountings and the use of the software Lido Studio during the trial period by various researchers and participants were successful. Despite some limitations, the lidos can help advance research on the non-visual responses to light over the coming years.

## 1. Introduction

When light impinges on our eye’s retina it triggers both visual and non-visual responses with profound influence on our behaviour, health and well-being. Lighting research on visual responses has a long tradition, and measuring devices are relatively easy to obtain. In contrast, lighting research on non-visual responses gained traction fairly recently (~20 years ago), when a critical photoreceptor, the intrinsically photosensitive retinal ganglion cell (ipRGC), was newly discovered in mice and then confirmed in humans.^1,2^ While these photoreceptors and the human body’s capacity to process light evolved under the natural light-dark cycle, human beings recently started spending most of the day indoors.^3–5^ In a building, daylight is filtered through window glass and supplemented or replaced with electric light. Thus, indoor light is usually lower in intensity, different in light distribution and spectral composition than natural light outdoors. Furthermore, artificial light extends the availability of light to the natural dark period, often negatively impacting human health in various ways.^6,7^

In the years that followed discovery of the ipRGC, there was no consensus on appropriate spectral sensitivity functions and metrics. Sometimes photometric quantities were used, despite them being deemed unsuitable for non-visual stimuli. Initial recommendations regarding the measurement of light with respect to non-visual responses were made in 2014 by a team of representatives from leading research groups.^8^ They were formalised into the international standard CIE S 026:2018 in 2018.^9^ Reacting to a perceived need for guidance, an international group of experts published recommendations for healthy indoor light exposure in 2022.^10^

Various wearable devices were developed by educational institutions and private companies to measure light exposure over time, that is, a person’s ‘light history’, and thereupon used in studies.^11^ They varied regarding their measuring point (wrist, chest or head), control (manual spot measurements or automatic time series measurements) and metrics among other things. A team from Lucerne School of Engineering and Architecture set out to develop a device featuring what they believed to address researchers’ needs, that is, a device that takes automatic measurements at preset intervals in the vicinity of a person’s face using the latest metrics.

## 2. Method

### 2.1 Conceptualisation and needs analysis

In 2018 a team from Lucerne School of Engineering and Architecture consisting of experts in the field of electronics, software development, industrial design and light and health started working on a user-centred concept for measuring optical radiation in the visible range (380 nm–780 nm). This multidisciplinary approach allowed for a holistic view on the development of the device, as decisions taken in one discipline influenced work to be done in other disciplines.

At the start of 2018, the team held semi-structured interviews with seven potential future users of such a device to find out their requirements, for example, light levels, wavelength range, recording intervals and additional desirable functionalities. In addition, the features and performance characterisations of devices used to investigate the non-visual responses to light were studied, for example, ActTrust (Condor Instruments, São Paulo, Brazil),^12^ Actiwatche (Philips Healthcare, Amsterdam, the Netherlands),^13^ LuxBlick (Technical University Ilmenau, Germany),^14^ ‘LightWatcher’ Data Logger (EUClock Programme, Vienna, Austria),^15^ Daysimeter and Daysimeter-D (Lighting Research Center of the Rensselaer Polytechnic Institute, Rensselaer NY, USA)^16^ and LYS (LYS Technologies LTD™, UK).^17^ In addition, assessments of such devices conducted and described by other researchers were analysed.^18,19^ Based on the information gathered from the interviews and the device analysis, a list with ‘must-have’ and ‘nice-to-have’ requirements was developed (Table 1) and subsequently used as a guide throughout the project of developing the Light-Dosimeters (lidos).

**Table 1:** Requirements used throughout the project

Element	*Must-have* requirements	*Nice-to-have* requirements
Device dimensions		
Size	⩽20 000 mm^3^	⩽16 000 mm^3^
Weight	⩽35 g	⩽20 g
Light sensor		
Wavelength range	380 nm –780 nm	In addition: UV detection
Dynamic range	Lower end: 1 lx Upper end: 20 klx	Lower end: <1 lx Upper end: >20 klx
Technical details		
Battery life	5–7 days	>7 days
Recharge	Cable	Induction
Memory size	512 MB	> 512 MB
Data transfer	Cable	Wireless
Recording interval	10 s	⩾1 s–freely selectable
Optic system	Cosine	Customisable field of view
Acceleration sensor	Tilt	In addition: Sideway
Ingress protection	IP20	IP54
Human-machine interface		
Measuring point	At the side of the head	In addition: At the chest
Event marker	Push-button	In addition: Acoustic feedback
Battery status indicator	RGB LED	In addition: Acoustic alert
Mounting	One type	Several different types
Software		
Operating system (OS)	Windows 7 or higher	In addition: Compatible with OSX
Open access data formats	csv, pdf and png/svg	In addition: Other data formats
Data storage	Local database	Cloud-based database
Metrics	Some traditional ones (e.g. *E_v_* and CCT) and some according to CIE S 026/E:2018^9^	Some traditional ones (e.g. *E_v_* and CCT) and all according to CIE S 026/E:2018^9^

### 2.2 Light sensor selection

**Figure 1:**
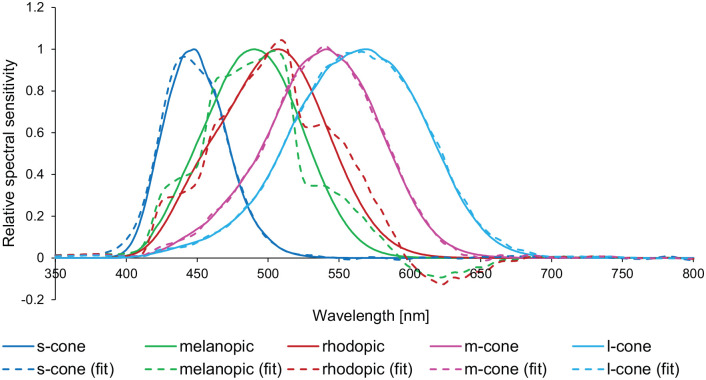
Approximations (dashed lines) of the five spectral sensitivity functions (solid lines) according to CIE S 026/E:2018 under CIE Standard Illuminant D65 [order of functions according to legend]

A market analysis led to the identification of six commercially available light sensors/mini-spectrometers as potential candidates for the future device. After a comparison of their properties, for example, wavelength range, resolution, size, price and energy consumption, the development kits of the most promising two were purchased, that is, a multichannel sensor and a spectral light sensor. Considering the results of the in-house evaluations and the advantages and disadvantages of each, the multichannel light sensor was viewed as the better option for the project. The spectral appraisal of its six optical channels covering the wavelength range 400 nm–1000 nm was supported by the METAS optics laboratory (Bern-Wabern, Switzerland). This multichannel light sensor was able to measure light levels just below 1 lx. Further value was seen in the shorter integration time of a multichannel light sensor at low light levels compared to that of a spectral light sensor. Furthermore, its lower energy consumption allowed for a smaller, lighter battery enabling the miniaturisation of the device. As the multichannel light sensor did not have the CIE spectral sensitivity functions according to CIE S 026/E:2018,^9^ ordinary least squares approximations were used with CIE Standard Illuminants A, D65, B1–B5, BH1, RGB1, V1 and V2. Figure 1 provides a graphic representation of the approximations under CIE Standard Illuminant D65 as an example. Due to daylight’s continuous spectrum, the team decided to use the transformation factors optimised for CIE Standard Illuminant D65 in the future device.

### 2.3 Preliminary work

**Figure 2:**
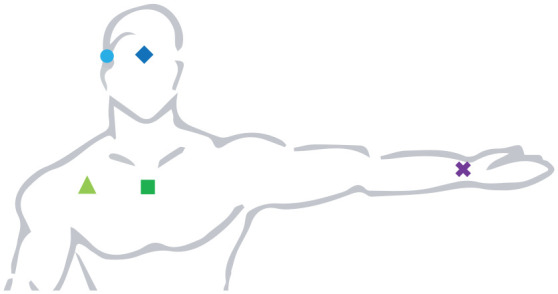
Measuring locations used in the data logger analysis: In the centre and at the side of the head, at the wrist, and in the centre and at the side of the chest

**Figure 3:**
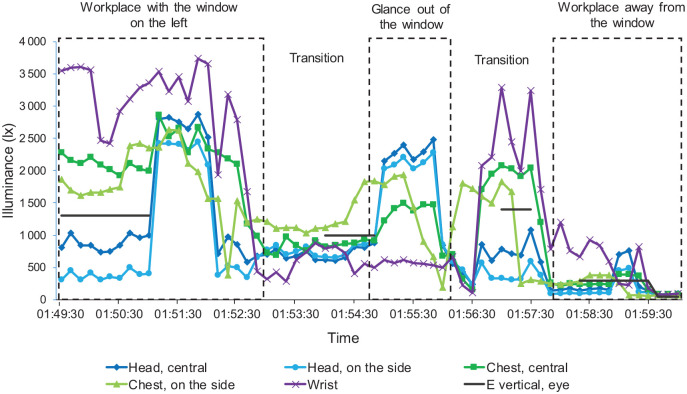
Example of measurements taken at different measuring locations for comparison in the data logger analysis

As a starting point for the design concept, the variation of measurements taken at different positions was tested. Illuminance was measured with modified light data loggers (HOBO UA-002-64, Onset, Bourne MA, USA), whose light sensors had been replaced with silicon PIN photodiodes (VEMD5510C, Vishay, Malvern, PA, USA). Two photodiodes were attached to a pair of glasses, one to a bracelet and two to magnetic silicone clips. This allowed measurements at five different positions on the participant’s body to be taken simultaneously: In the centre and at the side of the head, at the wrist, and in the centre and at the side of the chest (Figure 2). These measurements were then compared with measurements (vertical, at the eye) taken by an illuminance meter (T-10, Konika Minolta, Japan). The results confirmed findings from previous studies^20^ that the differences in the illuminance levels recorded were quite substantial for some settings. Figure 3 depicts the simultaneously taken measurements of the five photodiodes and the measurements taken by the illuminance meter in three different situations. The data analysis showed that the measurements taken at eye level most closely matched the illuminance meter measurements.

Despite the risk of a potentially lower user acceptance compared with a wrist or chest solution, the results convinced the team to develop a device capable of taking measurements at eye level, as they also take an account of participants’ head movements. This decision was also supported by experts in the field of light and health whom the team consulted. Measurements in the near-corneal plane are viewed as the best available proxy to retinal irradiation by most researchers.^21^ With regards to the exact position of the sensor, a solution at the side of a person’s face was favoured. The gain in accuracy from a solution with a light sensor in the centre of a participant’s face due to it being less likely covered by, for example, the hair was viewed as not sufficient to outweigh the potential lower user acceptance due to the prominent spot.

### 2.4 Hardware

After this initial phase of gathering information, the team started to develop the various elements of the device.

In addition to the light sensor, key hardware components on the printed circuit board are:

- an acceleration sensor, which tracks the tilt of the device, that is, the angle of a participant’s line of sight, if the lido is attached to a spectacle frame worn,
- a push button, which can be used to mark events as pre-defined by the lead investigator, for example, the actual beginning and end of an experiment, and ad hoc events that need to be documented and
- an RGB LED, which indicates a device’s battery status once activated.

A bespoke casing was designed to house the electronics, a rechargeable battery, an infrared cut-off filter (BOROFLOAT^®^) and a diffuser (uncoated white opal glass). The infrared cut-off filter, which mainly blocks long wavelength optical radiation, and the diffusor, which produces uniformity of the incoming optical radiation, both cover the light sensor at the front of the device (Figure 5). The use of these two components means that the device is unable to measure at light levels just below 1 lx, as the light sensor alone was able to do. Instead, the recommended measurement range of the device starts at 5 lx.

**Figure 4:**
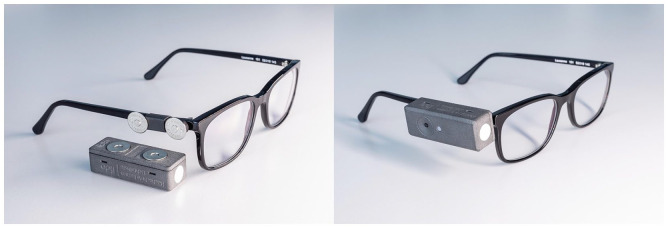
A Light-Dosimeter (lido) unattached and attached to a spectacle frame

**Figure 5:**
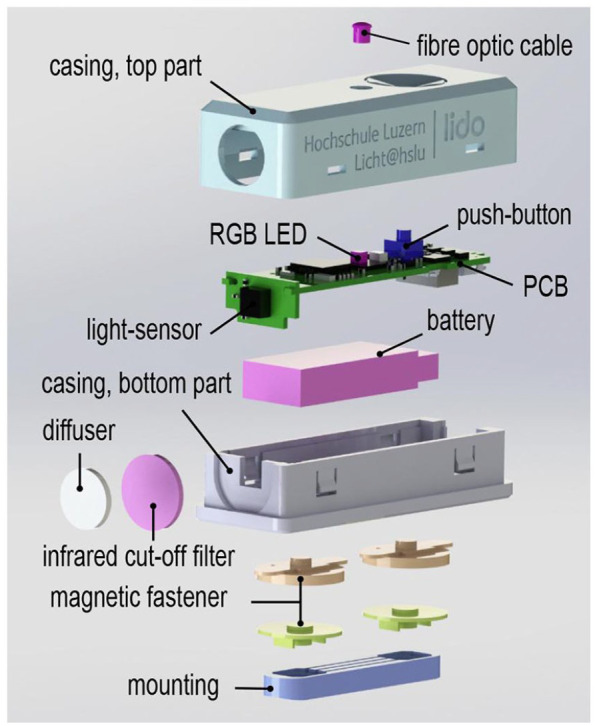
Exploded view of the key components used in a Light-Dosimeter (lido)

After an assessment of different 3D printing materials, the decision was taken to use Polyamid 12 (PA 12) for the casing. As this material is opaque, a 3-mm fibre optic cable was used to make the light of the RGB LED visible on the outside of the casing. A partition surrounding the RGB LED reduces potential spill light inside the casing. However, its flashing light does not interfere with the recordings in any case, as it occurs just after a measurement. In addition, a bespoke mounting was designed, so that the device can be taken off during a recording if required (see the left of Figure 4). This mounting (also 3D printed with PA 12) can be attached to most spectacle frames and other frames, for example, those of eye tracking devices. The key components used in a lido can be seen in Figure 5 in an exploded view.

The initial idea to make the recording interval freely selectable was dropped after some investigations, as it would have exceeded the available resources. Instead, the lidos were programmed to take measurements in 10-s intervals. That frequency was decided upon after consultations with various researchers and taking into account the volume of data each measurement generates and the associated energy requirements. Each measurement has a unique timestamp. The specification of the final in-house manufactured devices is summarised in Table 2.

**Table 2:** The technical specifications of a Light-Dosimeter (lido)

Element	Details
Size of the casing	58 mm × 20.6 mm × 16 mm
Weight of the device	~27 g
Battery life	~7 days
Battery charging time	~2 h
Memory size	~300 days
Wavelength range	VIS 380 nm–780 nm
Dynamic range	~5 lx–100k lx
Angular response	Close to cosine (f2 approx. 5%)
Interface	Micro USB
Ingress protection	IP20

### 2.5 Calibration

Each lido was calibrated using the following equipment:

- a spectroradiometer (specbos 1201; JETI Technische Instrumente GmbH, Germany; calibration certificate serial number 2911599),
- an integrating sphere (AvaSphere-200; Avantes BV, the Netherlands),
- a reference light source (64634 HLX; Osram GmbH, Germany),
- an infrared cut-off filter (item number 53-710; Edmund Optics GmbH, Germany) and
- a personal computer/laptop running a National Instruments LabVIEW programme.

**Figure 6:**
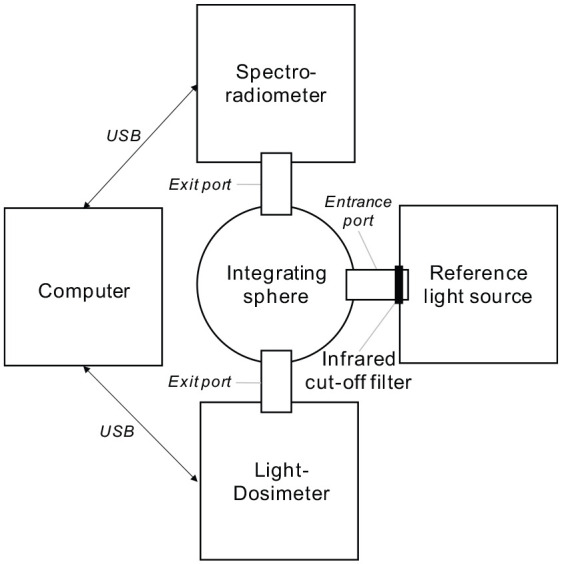
Block diagram of the setup used to calibrate the Light-Dosimeters (lidos)

Figure 6 shows a block diagram of the calibration setup. For the calibration, a lido was coupled with a custom-made 3D printed mounting to one of the integrating sphere’s exit ports the spectroradiometer was coupled directly to another one. The lidos were calibrated at room temperature. The infrared cut-off filter at the exit port of the reference light source prevented parts of the calibration setup from heating up. A dark room was not required, as checks had confirmed that the setup used let only a negligible amount of light enter the integrating sphere. The reference light source was given time to heat up. Its stability was checked by measurements taken with the spectroradiometer. The measurements taken with the spectroradiometer and each lido during the calibration were not quite simultaneous due to the lidos taking measurements at a preset interval.

After the calibration, each lido ran through a series of tests. First, the performance of the light sensors was checked against simultaneous measurements taken by a spectroradiometer (specbos 1201; JETI Technische Instrumente GmbH, Germany) under an incandescent light source, a white LED, a white fluorescent light source and daylight. Then the devices’ tilts were checked at −90°, 0° and +90°, that is, the light sensor pointing vertically downwards, horizontally and vertically upwards, respectively.

### 2.6 Software

**Figure 7:**
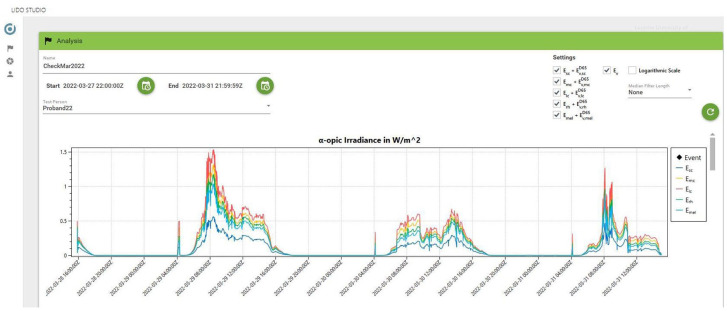
Screenshot of the data analysis view in Lido Studio: Selection of timeframe and participants (top left), setting options, for example, metrics, filtering and logarithmic scale (top right) and example graphic displaying α-opic irradiance (in W/m^2^) over a 3-day window

The software package Lido Studio was developed using Microsoft Visual Studio. It runs on a computer with Windows OS. On its graphical user interface, the lead investigator enters the details of the planned experiment (e.g. its time frame and participants’ details) and activates the lido to be used. After collecting data, the investigator deactivates the lido and downloads the data in Lido Studio, which stores it in a local database, that is, the data sovereignty lies with the researcher. Using the software, initial analyses can be run to display charts and plots instantaneously (Figure 7). The user can adjust the view, for example, deselecting certain metrics, choosing specific time frames and selecting a logarithmic scale. The selections made on screen can be turned into a PDF report, the plots saved as images and the data exported as a comma-separated values (CSV) file for further analyses.

The Lido Studio provides time series data for the event marker and the following metrics:

- α-opic irradiance ( $E_{\alpha }$ ) according to CIE S 026/E:2018,^9^
- α-opic equivalent daylight (D65) illuminance ( $E_{v,\alpha }^{D65}$ ) according to CIE S 026/E:2018,^9^
- illuminance ( $E_{v}$ ),
- correlated colour temperature (CCT) and Duv^22^ and
- tilt angle (between −90° and +90°).

### 2.7 Initial measurements

Once a first set of lidos had been produced, they were tried out by three different parties using different approaches. Several lidos were tested with regards to their spectral and geometric properties in the METAS optics laboratory (Bern-Wabern, Switzerland) in early 2021.

In spring and early summer 2021, the team from Lucerne School of Engineering and Architecture used the lidos to investigate light exposures over several days in real-life settings by wearing the devices themselves, having other people wear them, and by taking measurements at specific locations.

To generate preliminary data and demonstrate a possible scientific application of the lidos, team members at the Centre for Chronobiology (Basel, Switzerland) deployed a lido as part of an ongoing investigation of the spectral determinants of steady-state pupil size under real-world (light) conditions during daytime in summer 2021. Previous findings suggest that steady-state pupil size is primarily determined by the melanopsin-encoded signal under such continuous light levels.^23–25^ Three healthy participants were involved in the tests as an ad hoc sample: two female subjects (24 and 25 years old) and one male subject (57 years old). The corresponding datasets were labelled according to the participants’ ID ‘SP025’, ‘SP029’ and ‘SP033’. Specifically, measurements taken by a lido were compared with those taken by a small spectroradiometer (STS-VIS; Ocean Insight, USA). This was done in a field-compatible ambulatory setup in which the lido was mounted on an eye tracker frame (Pupil Core; Pupil Labs, Berlin, Germany), and the STS-VIS was mounted on a participant’s forehead facing forward (at a 15° angle below the horizontal). The data were collected in 10-s intervals and binned into 1-min averages of melanopic irradiance to account for slight shifts in the sample period between the two devices (max. 3 s). Testing took place across three experimental sessions of an approximate 1-h experiment protocol which started with a 10-min dark adaptation and continued in varying indoor and outdoor light conditions by daylight. The different light situations in the experiment each typically lasted for ⩾ 1 min. Values below 5 lux (corresponding to ⩾3 mW·m² melanopic irradiance in the data) were excluded from the comparison as they were below the recommended measurement range of the lido (Table 2). Ethical approval had been granted from the cantonal ethics commission: Ethikkommission Nordwest- und Zentralschweiz, project ID 2019-01832.

## 3. Results

### 3.1 Spectral and geometric properties

**Figure 8:**
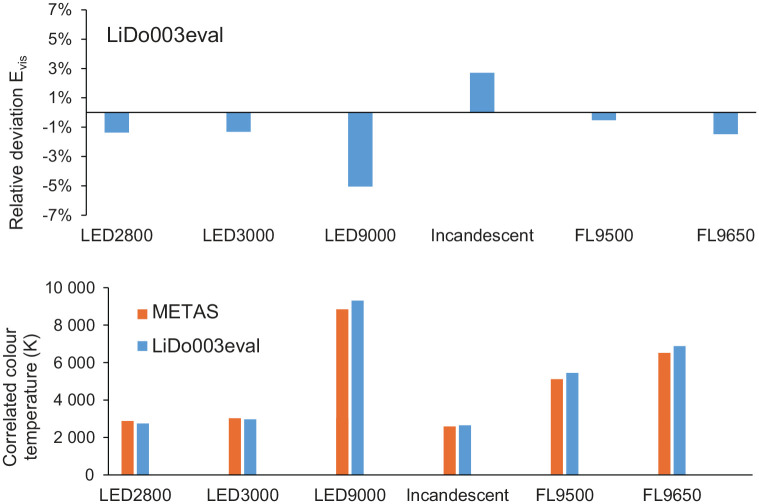
A Light-Dosimeter’s (lido’s) relative deviation of illuminance, CCT comparison

**Figure 9:**
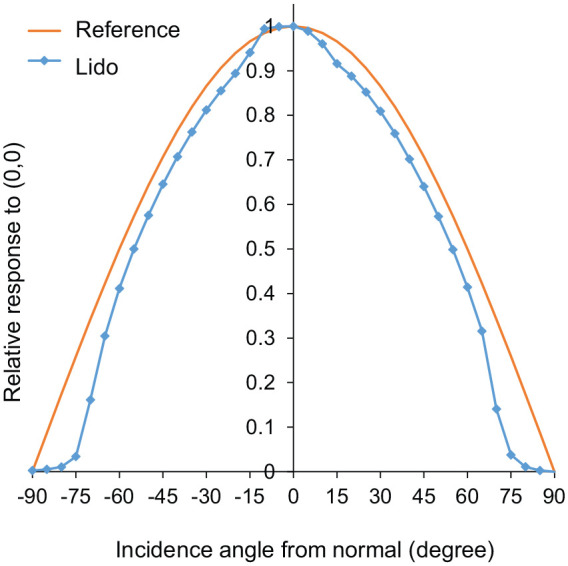
A Light-Dosimeter’s (lido’s) angular response (left/right) compared to an ideal cosine response

The spectral and geometric tests at the METAS optics laboratory (Bern-Wabern, Switzerland) found that the deviations of α-opic equivalent daylight (D65) illuminance and α-opic irradiance were in the order of 10% for all white light sources and the deviation of illuminance was less than 5% for white light sources (Figure 8). Furthermore, the deviation of CCT was <100 K for CCTs below 4500 K and <400 K for CCTs above 4500 K. Figure 8 also depicts a lido’s absolute CCT values measured in comparison with those from a reference spectroradiometer, which is directly traceable to the International System of Units (SI) through METAS, and its geometric properties, that is, its angular response in comparison with a cosine response (Figure 9).

### 3.2 Anecdotal evidence from trial period

The trial period offered team members from Lucerne School of Engineering and Architecture (Horw, Switzerland) a chance to familiarise themselves with the final version of the lido and the software package Lido Studio. Key observations were lower than expected indoor light levels at workplaces, when measured in the near-corneal plane, and negative average tilt angles. Another key learning was the benefit of keeping a diary. The so collected background information complemented the recordings and helped to better understand the data. The following entries are suggested as a minimum: date, time, event marker pressed (yes/no) and notes, for example, in case of a change of location and removal of the device. It is recommended that a participant is given clear instructions regarding the handling of the device, the use of the event marker and the keeping of the diary. Furthermore, they should be provided with some background information about the non-visual responses to light, as wearing a lido in real-life settings led to a lot of questions and interesting conversations. Children as young as 2 years old noticed them and asked about their purpose.

### 3.3 Pupil response measurements and validation with commercial spectroradiometer

**Figure 10:**
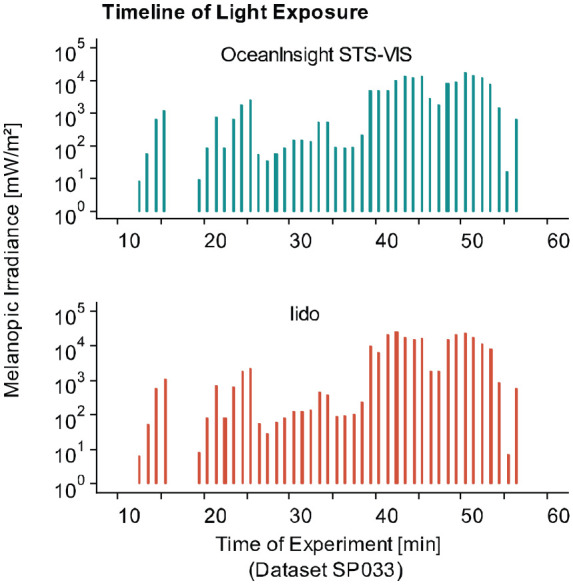
Melanopic irradiance measured by a spectrometer (Ocean Insight STS-VIS) and a Light-Dosimeter (lido) over time from dataset SP033

**Figure 11:**
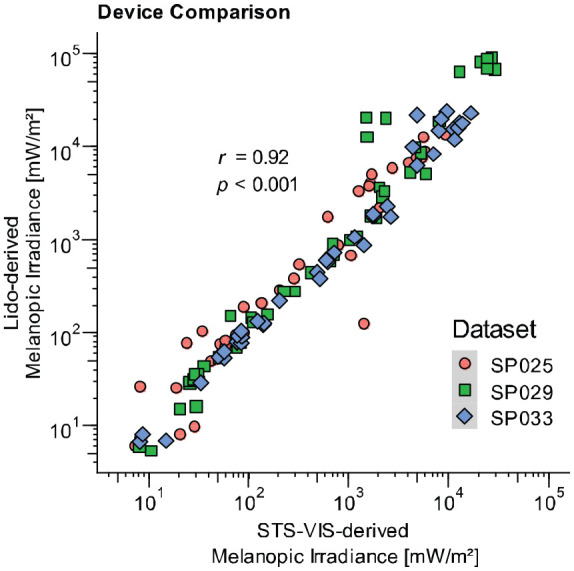
Device comparison using three datasets (SP025, SP29 and SP033)

**Figure 12:**
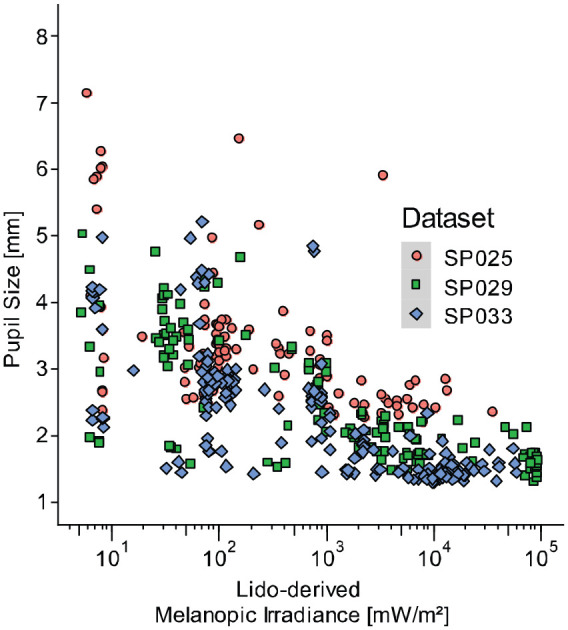
Relating melanopic irradiance levels derived from a Light-Dosimeter (lido) to pupil diameter for three datasets (SP025, SP29 and SP033)

Figure 10 displays the timeline of near-corneal light exposure in one experiment session (SP033) of an ongoing investigation of the spectral determinants of steady-state pupil size under real-world conditions at the Centre for Chronobiology (Basel, Switzerland). A small commercial spectroradiometer (STS-VIS; Ocean Insight) and a lido show homogenous variations in melanopic irradiance across the experiment session. Samples below the recommended measurement range (<5 lx, Table 2), mostly stemming from the 10-min dark adaptation, were excluded from the comparison. As depicted in Figure 11, 1-min averages of melanopic irradiance derived from the lido were highly correlated with those derived from the small commercial spectroradiometer (*r* = 0.92, *p* < 0.001, Pearson coefficient). These results indicate that the measurements of the two devices were comparable. Additionally, near-corneal melanopic irradiance measured by the lido was related to pupil size measurements derived from a Pupil Labs eye tracker (Pupil Core; Pupil Labs, Germany). The results are illustrated in Figure 12. Notably, within-subject pupil diameter varies substantially at low irradiance levels, displaying much more stability at high levels. In addition, the data show distinct dose–response curves for each subject, indicating that variation in steady-state pupil size under continuous light conditions can successfully be described as a function of near-corneal melanopic irradiance measured by a lido.

## 4. Discussion

The user-centred approach taken during the development led to a holistic solution consisting of three parts: the devices, mountings and software Lido Studio. The results achieved during the trial period look promising. Overall, the devices take quite accurate measurements. Even more, they take them in the near-corneal plane, which is deemed more suitable to investigate the non-visual responses to light than, for example, the chest or wrist. The handling of the devices and mountings and the use of the software Lido Studio during the trial period by various researchers and participants went well.

However, there are some limitations to the lidos’ use. They are less suited for evening and night studies, as measurements below 5 lx should be excluded. Furthermore, participants with full vision might not like the idea of wearing glasses for a long period of time affecting the compliant use and therefore data quality. And lastly, the lidos can only cover some of the recommendations for reporting light exposure in studies investigating non-visual responses to light^26^; others such as the spatial distribution of the stimulus need to be characterised with the help of other devices.

## 5. Conclusion

Though the lidos have some limitations, they still supply the community investigating the non-visual responses to light with a new tool. Researchers are provided with time series data from participants’ near-corneal plane light exposure using metrics from CIE S 026:2018 to test their hypotheses. Current recommendations on daytime light exposure can thus be validated. In the medium to long term, the lidos’ contribution might lead to an improved understanding between an individual’s light exposure and the physiological, psychological and behavioural responses to light and a healthier ‘light hygiene’ in modern society.
